# Absence of Arrhythmogenicity with Biphasic Pulsed Electric Fields Delivered to Porcine Airways

**DOI:** 10.1007/s10439-023-03190-5

**Published:** 2023-04-25

**Authors:** Glenn R. Meininger, Robert E. Neal, David W. Hunter, William S. Krimsky

**Affiliations:** 1 Cardiology, MedStar Union Memorial Hospital, Baltimore, MD USA; 2 Galvanize Therapeutics, 1531 Industrial Road, San Carlos, CA 94070 USA

**Keywords:** Arrhythmia, Cardiac synchronization, ECG, Cardiac gating, ECG-sync, Electroporation

## Abstract

Pulsed electric field (PEF) technologies treat many types of tissue. Many systems mandate synchronization to the cardiac cycle to avoid the induction of cardiac arrhythmias. Significant differences between PEF systems make the assessment of cardiac safety from one technology to another challenging. A growing body of evidence suggests that shorter duration biphasic pulses obviate the need for cardiac synchronization, even when delivered in a monopolar fashion. This study theoretically evaluates the risk profile of different PEF parameters. It then tests a monopolar, biphasic, microsecond-scale PEF technology for arrhythmogenic potential. PEF applications of increasing likelihood to induce an arrhythmia were delivered. The energy was delivered throughout the cardiac cycle, including both single and multiple packets, and then with concentrated delivery on the t-wave. There were no sustained changes to the electrocardiogram waveform or to the cardiac rhythm, despite delivering energy during the most vulnerable phase of the cardiac cycle, and delivery of multiple packets of PEF energy across the cardiac cycle. Only isolated premature-atrial contractions (PAC) were observed. This study provides evidence that certain varieties of biphasic, monopolar PEF delivery do not require synchronized energy delivery to prevent harmful arrhythmias.

**Supplementary Information:**

The online version contains supplementary material available at 10.1007/s10439-023-03190-5.

## Introduction

Pulsed electric field (PEF) therapies use brief applications of electrical energy to alter the transmembrane potential of cells and organelles. This disrupts membrane integrity, disrupting cellular homeostasis and initiating a cascade of biochemical processes which induce different forms of cell death including necrosis, apoptosis, aponecrosis, necroptosis, and/or pyroptosis [18].

PEF therapies are not dependent on thermal processes, providing the potential to kill cells within a volume of tissue *in vivo* without altering the extracellular matrix. This facilitates the preserved function of critical and sensitive anatomic structures such as the major vasculature [15], luminal systems such as the common bile duct [25], and tissue structures such as the pleura [7, 30]. In this context, PEF therapies may offer a superior safety profile relative to other focally ablative modalities [8, 10] and radiation [28]. Clinically, PEF is being applied in a variety of disease states with increasing regularity, including cancer, heart disease, and lung disease [25, 29, 31, 32].

Variations exist between clinical PEF systems, including differences in waveform parameters and delivery polarity (i.e., bipolar or monopolar). In bipolar electrode configurations, energy is delivered between effector devices placed within, or adjacent to, the targeted environment. Conversely, monopolar systems use a single end-effector to deliver energy to the targeted location with a remote dispersive electrode serving as the electrical return. The dispersive electrode is of sufficient surface area to distribute the PEF energy broadly enough that no treatment effects are encountered at its location.

The energy may be delivered as square-wave DC-type waveforms—referred to as monophasic—with each pulse lasting tens of microseconds [29]. Long monophasic pulses produce strong cellular lethality but are typically accompanied by pronounced muscle contraction because of action potential generation in motor neurons and skeletal muscle. These treatments require neuromuscular paralytic. Even then, visible patient contractions remain [2]. Alternatively, much shorter duration monophasic treatments can be delivered, which use nanosecond-scale electric (nsPEF) pulses [3]. While this reduces or eliminates muscle contraction, there is significant compromise to cellular lethality, requiring tens of thousands of volts per centimeter to induce cell death. In a third configuration, a series of short (≤ 10 µs) DC-type pulses can be compiled with alternating polarity, which result in significantly reduced muscle contraction with only a mild reduction in cellular lethality [1, 16].

An important consideration regarding clinical use of PEF energy is its potential to stimulate cardiac tissue and interfere with the normal cardiac cycle. Several variables impact the arrhythmogenic potential of any given PEF therapy including the location, extent, and timing of this stimulation [5]. Many PEF technologies reduce arrhythmogenesis by synchronizing PEF delivery to the cardiac refractory period (i.e., ST interval) [5, 22, 29, 33].

Notably, shorter pulse durations reduce the probability of inducing an arrhythmia [14]. It is well understood that stimulation of excitable tissues follows a strength–duration curve. Lawo et al. characterized this relationship for monophasic pulses and showed that not only does the stimulation threshold increase with shorter pulse duration, but the ventricular fibrillation (VF) induction threshold also increases exponentially as the pulse width decreases [12]. They note that since the chronaxie for stimulation is more than an order of magnitude less than that of fibrillation, shorter pulses are much less likely to induce VF. Common IRE-type ablation pulses (70–100 µs) fall within the ventricular fibrillation induction range of these strength duration curves for the typical 1–3 kV pulses. Conversely, PEFs of 1–5 µs duration and 1–3 kV exceed the stimulation threshold but remain well below the VF induction threshold.

Additionally, biphasic waveforms are generally less arrhythmogenic than monophasic waveforms due to two factors: (1) Biphasic waveforms have higher thresholds for stimulation and VF induction than monophasic waveforms [21, 23], and (2) biphasic waveforms result in more homogenous tissue polarization, which reduces the likelihood of post-shock voltage gradients initiating an arrhythmia [24].

Consequently, short-duration, biphasic PEF waveforms may reduce or eliminate potential arrhythmogenesis. However, since the induction of cardiac arrhythmia is highly dependent on the 3-dimensional cardiac substrate (including cardiac fiber anisotropy), heterogeneous conductivity (including in tissue planes between the electrical source and the myocardium), and the complex spatiotemporal interaction of the electric field produced therapeutically with the intrinsic electrical wavefronts in the heart, any electrical therapy must be evaluated *in vivo* to determine its true arrhythmogenic potential. Herein, we assess the arrhythmogenic potential of a specific short-duration biphasic PEF therapy by testing it in a highly arrhythmogenic animal model and purposefully applying the therapy at the most vulnerable therapeutic anatomical locations and times within the cardiac rhythm.

This article describes the investigation into the potential risk for the RheOx® system (Galvanize Therapeutics, CA, USA), a biphasic PEF waveform delivered in a monopolar fashion, to induce arrhythmias in a porcine model. The system was clinically evaluated with delivery synchronized to the cardiac cycle in a 30-patient trial published by Valipour et al., which reported no observed arrhythmias related to energy delivery [31], but the arrythmia risk for asynchronous delivery has not been previously evaluated.

## Materials and Methods

### Theoretical Evaluation of Arrhythmogenicity Likelihood

Numerical simulations offer a valuable tool for representing electrical tissue changes in response to PEF therapies [17]. The cardiac safety of PEF therapies depends on several factors, including the electrical pulse duration, the applied voltage, and the distance to the heart. This analysis investigated these factors and their impact on two cardiac phenomena: cardiac activation and the induction of fibrillation. Comsol Multiphysics 5.4 (Comsol, Sweden) was used to model the voltage distribution and electric fields from various applied voltages.

#### Geometry

The electrode was modeled as a series of five rings expanded within a 1 cm diameter airway. Each ring was a 1 mm width boundary in contact with the airway circumference and separated by 1 mm along the length of the airway. Epithelial and submucosal layers were considered 0.35 mm thick and cartilage 0.7 mm thick, consistent with approximate dimensions of porcine airway tissue layers. The airway region was placed within a 20 cm diameter and 40 cm long lung parenchymal region, with a 5 cm diameter dispersive plate electrode placed at the end.

#### Tissue Electrical Properties

The airway interior was modeled as air, with an electrical conductivity of 1 ×10^–7^ S/m, while epithelial and cartilage layers each had electrical conductivity of 0.362 S/m [9]. The lung parenchyma was considered to exhibit dynamic electrical conductivity as a function of electrical field, as described previously [19, 20]. This resulted in the electrical conductivity function of

1 $$\sigma (E) = {\sigma }_{0}+A\times {e}^{-{e}^{\frac{-(E\,-\,B)}{C}}},$$

where *σ*_0_ is the baseline electrical conductivity of inflated lung at a frequency consistent with 1 µs pulse duration (0.126 S/m), which increases with electric field exposure to approach the electrical conductivity of inflated lung at frequencies in the β-dispersion realm (200 MHz, 0.335 S/m) [9]. The 1 µs pulse duration was selected since it is within the typical range of biphasic PEF waveforms. The values for B and C provide the center and ± distance to the inflection points along the curve of 400 and 125 V/cm, respectively, which produces a conductivity function that increases as electric field exposure increases from approximately 250 to approximately 1000 V/cm (Supplemental Fig. 1). This range and curve shape are consistent with experimental data [20]. Only electrical effects were modeled in the simulation, as this is the most influential in arrhythmia risk.

#### Numerical Simulation Conditions

The geometry was meshed into 1.24 × 10^6^ elements (“extra fine” physics-controlled mesh). The external boundaries were considered electrically insulating, while interior boundaries followed current conservation. The dispersive pad boundaries were set to ground. The electrode rings were evaluated in a parametric study, with the applied electrical voltage evaluated from 1000 to 3000 V in 1000 V increments. The electrical voltage as a function of distance from the electrode rings in the tissue was determined.

#### Theoretical Arrhythmogenicity Threshold

Both cardiac stimulation (i.e., pacing) and arrhythmia induction are voltage and pulse duration-dependent processes, and those strength–duration relationships were extracted from Lawo et al. who described them for approximately 90 µs–50 ms monophasic pulses [12]. Notably, the thresholds to pace the heart and to induce arrhythmia diverge sharply below pulse widths of ~ 1 ms. Data from monophasic pulses were extrapolated since the most robust PEF-related arrhythmia literature was conducted with monophasic pulses, and biphasic waveforms have been previously shown to have higher stimulation and VF induction thresholds, meaning monophasic pulses represent a worst-case scenario [21, 23]. The data from Lawo et al. were extrapolated by fitting the VF induction and stimulation curves with a power law relationship as depicted in Supplemental Fig. 2. These data were used to infer the likelihood of inducing either cardiac effect as a function of applied voltage and distance from the heart for various pulse durations between 1 and 100 µs.

### In Vivo Porcine Experiments

In this IACUC approved study, four swine (44.3–63.3 kg) received PEF energy. Electrocardiograms (ECGs) were recorded from a data acquisition module (DAQ) connected to an Ivy 7600 cardiac monitor (Ivy Biomedical, CT, USA) and the PEF generator. The cardiac monitor outputs the ECG waveform and a trigger pulse that corresponds in time to the R-wave of ventricular depolarization (the R-trigger), which allows precise control of PEF delivery timing relative to cardiac depolarization. The cardiac monitor was connected to the subject with a 4-lead cable. The DAQ concurrently recorded the ECG signals, R-triggers, and the delivery of PEF energy, enabling review of PEF energy delivery relative to the pig cardiac rhythm.

ECGs were recorded continuously during all experiments and were reviewed by a board-certified electrophysiologist to interpret any changes in cardiac rhythm or ECG waveform resulting from PEF delivery. ECGs were screened for signal saturation, an impact on R–R timing interval (heart rate), atrial activation or atrial arrhythmias, and ventricular activation or ventricular arrhythmias including tachycardia, bradycardia, and fibrillation. Any other ECG incidental findings were also noted.

C-arm fluoroscopy visualized the basket catheter position. Computed tomography (CT) images of the porcine chest were performed to determine airway-cardiac proximity.

#### PEF System Background

**Fig. 1:**
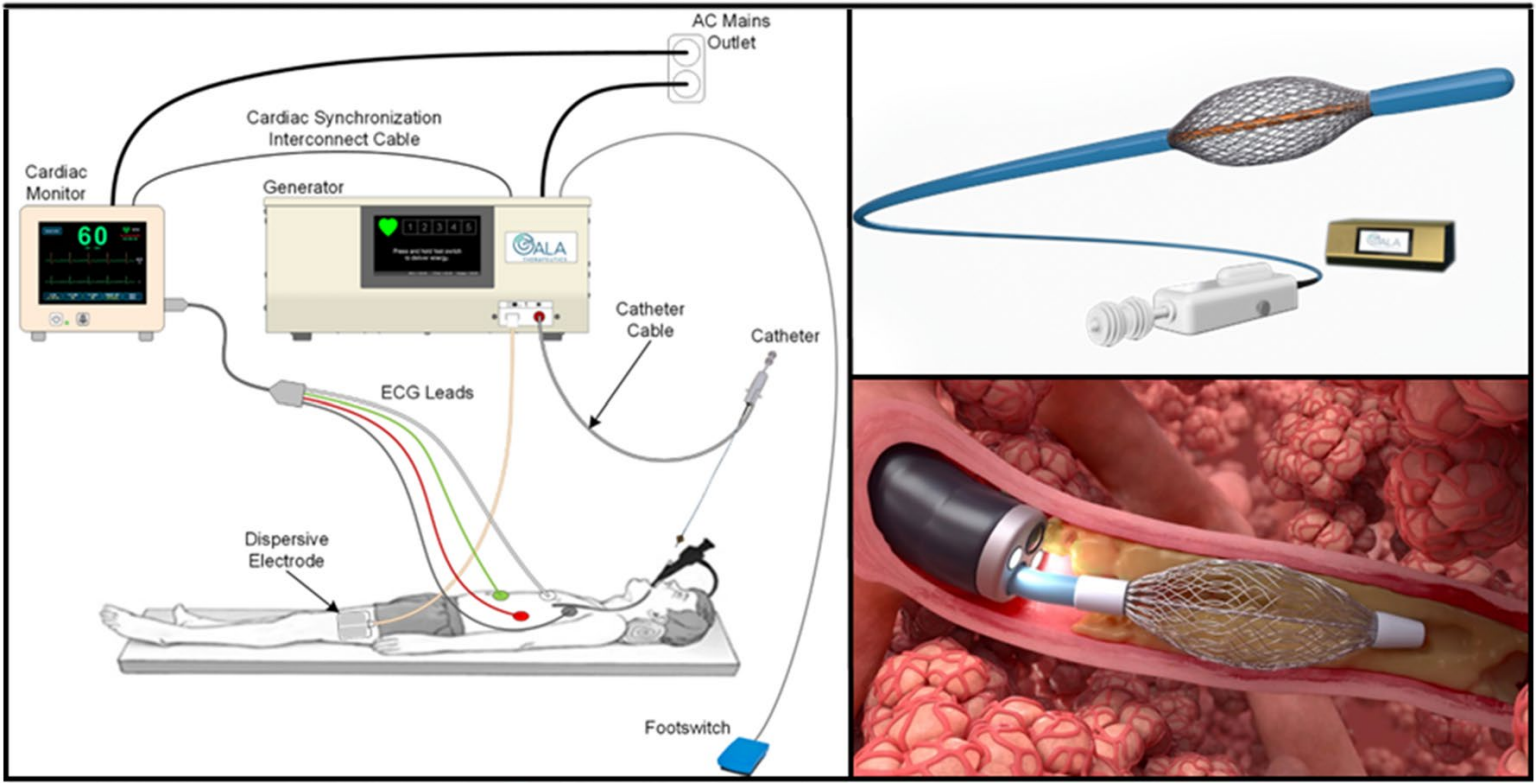
PEF system setup: A PEF generator connected to a cardiac monitor and expandable basket electrode was used to deliver energy. The energy is returned through a dispersive electrode

The system evaluated in this study is a PEF technology targeting the treatment of chronic bronchitis. The monopolar system (Fig. 1) consists of a PEF generator, footswitch, expandable basket electrode, and a large dispersive electrode placed at a distant location on the body [31]. The generator delivers a series of packets of PEF energy, with each packet composed of multiple biphasic pulses [32]. The basket electrode–tissue interface has markedly smaller surface area (0.67–1.30 cm^2^, depending on airway diameter) relative to the dispersive electrode (approximately 100 cm^2^).

Three experiments were conducted to test worst-case timing of PEF in directed attempts to deliberately induce cardiac arrhythmias: a single packet of PEF delivered at timed points over the entire cardiac cycle (Full ECG Sweep), a single packet delivered at tightly timed intervals over the most vulnerable T-wave portion of the cardiac cycle (High-Resolution T-wave Delivery), and multiple packets arranged together and delivered over the entire cardiac cycle (Multiple Packets). All studies used a generator with custom software to allow for precise timing of energy delivery relative to the R-trigger. PEF energy was delivered in various positions in the airway tree, including distal and proximal sides of both the left and right lungs to account for positional and anatomical variability around the heart.

#### Full ECG Sweep

In two pigs, a single packet of PEF was delivered. The generator sequentially extended the R-trigger delay interval before PEF delivery in 25 ms increments. This resulted in PEF delivery over all phases of the cardiac cycle. One packet was delivered every 5 heartbeats to allow observation of the cardiac response before the next packet delivery.

#### High-Resolution T-Wave Delivery

In a third pig, a single packet was delivered at higher resolution (10 ms increments) to cover the entire duration of the anticipated vulnerable region of the T-wave. The T-wave is recognized as the portion of the ECG most susceptible to arrhythmia since the tissue at that time has differing degrees of ventricular repolarization, which can result in unidirectional block and arrhythmia induction when stimulated [11]. One packet was delivered every 5 heartbeats to facilitate interpretation of the occurrence of changes to ECG and cardiac rhythm from the PEF treatment. The entire T-wave sweep was repeated at the clinical PEF dose, as well as a dose that delivered 44% more energy than the clinical dose.

#### Multiple Packets

Most PEF therapies deliver a bundle of packets to accumulate cellular injury and increase cell death beyond what would be produced by delivery of a single packet. To evaluate cardiac safety in the context of multiple-packet deliveries, a fourth pig was used to investigate whether compounding PEF packets, delivered asynchronously, could induce arrhythmia when a single PEF packet could not.

The generator was set to deliver a sequence of 5 or 10 packets in a bundle. PEF bundle delivery was started at different times throughout the ECG waveform with a resolution of 40 ms to ensure adequate probing of all potentially vulnerable regions. Two trials had a resolution of 80 ms. The cadence of packets within each bundle was also varied, to deliver packets either quickly (5 Hz/300 ppm), slowly (0.66 Hz/40 ppm), or at a rate near the intrinsic heart rate of the animal (roughly 1.2 Hz/70 ppm). As with the prior experiments, ECGs were recorded for 5 beats following delivery of the bundle of packets to determine the presence of induced arrhythmias.

## Results

### Theoretical Evaluation

**Fig. 2:**
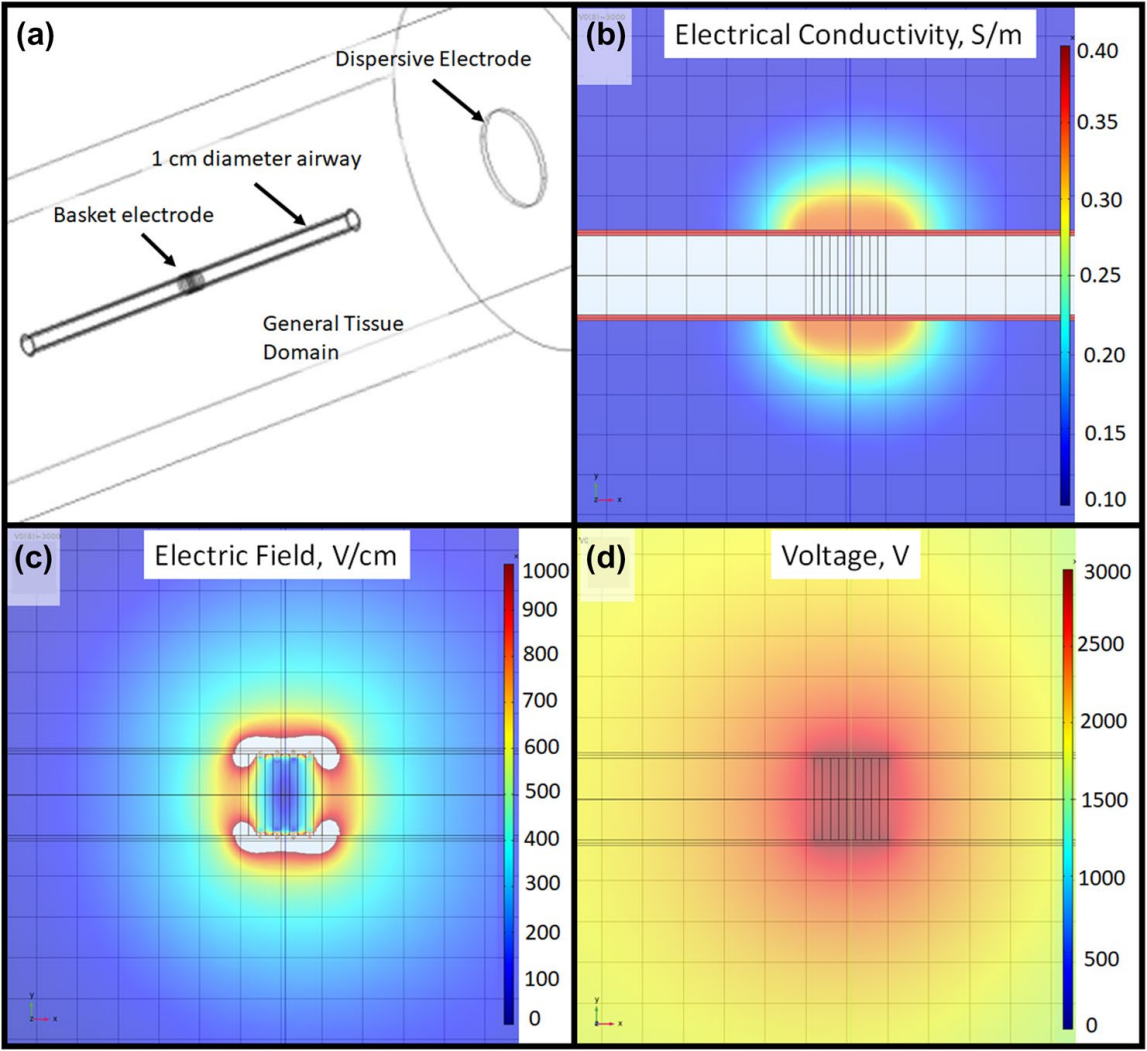
Numerical Simulation representative results. (**a**) Numerical simulation geometry. (**b**–**d**) Cross-sectional view of electrical conductivity, electric field, and voltage distributions into simulated lung parenchymal tissue. Grid = 5 mm

The geometry used in the numerical simulation, as well as representative distributions of electrical conductivity, electrical field, and electric voltage for a 3000 V pulse is shown in Fig. 2.

Dynamic electrical conductivity invoked a subtle decrease in tissue impedance with higher applied voltages due to a greater volume of tissue experiencing a PEF-induced increase in electrical conductivity (Supplemental Fig. 1).

**Fig. 3:**
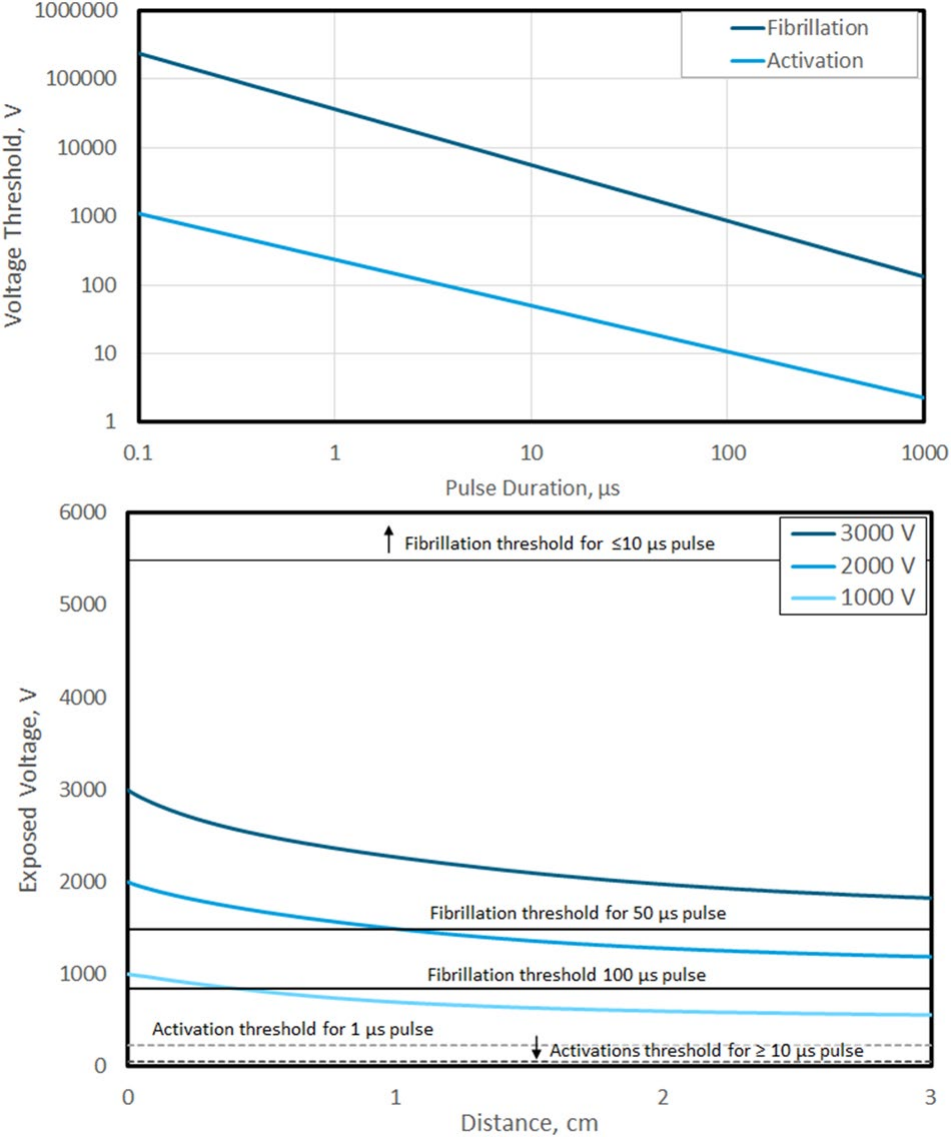
PEF cardiac effect sensitivity and relation to a basic treatment. (Top) Activation and fibrillation voltage thresholds as a function of pulse duration for a single monophasic pulse. (Bottom) Numerical simulation tissue voltage exposure for a basic monopolar basket electrode in the airway with different applied voltages. Activation and fibrillation voltage thresholds for selected pulses are superimposed, conveying the low activation threshold for all pulse durations, but very high fibrillation thresholds for pulses ≤ 10 µs

The top panel of Fig. 3 shows how the extrapolated voltage thresholds for cardiac stimulation and fibrillation induction are separated by approximately two orders of magnitude, and decrease logarithmically with increasing monophasic pulse duration. Although activation energies for pulses greater than 1 µs are less than 250 V, the voltages required to induce fibrillation for 0.5, 1, 10, 50, and 100 µs duration are much higher, decreasing from 62.6 to 35.6, 5.48, 1.48, and 0.843 kV, respectively. This highlights the high sensitivity of cardiac arrythmia to pulse duration.

The data from the numerical simulations were combined with the extrapolated threshold curves to produce the bottom panel of Fig. 3. In this figure, electric voltage decay in tissue over 3 cm at varying applied voltages is overlaid with the thresholds for activation and fibrillation at different monophasic pulse durations. From this figure, all applied voltages exposed the tissue to activation energies out to 3 cm deep. Furthermore, the voltage to cause fibrillation for 50 and 100 µs duration pulses was exceeded within 1 cm for all pulses when the applied voltage was above 2 kV. Conversely, for pulse durations ≤ 10 µs, *none* of the applied voltages resulted in tissue exposed to voltages capable of inducing fibrillation at any distance. These data indicate a substantial safety margin against cardiac arrhythmia induction for PEF treatments when monophasic pulse duration are 10 µs or less.

### Experimental Evaluation

**Fig. 4:**
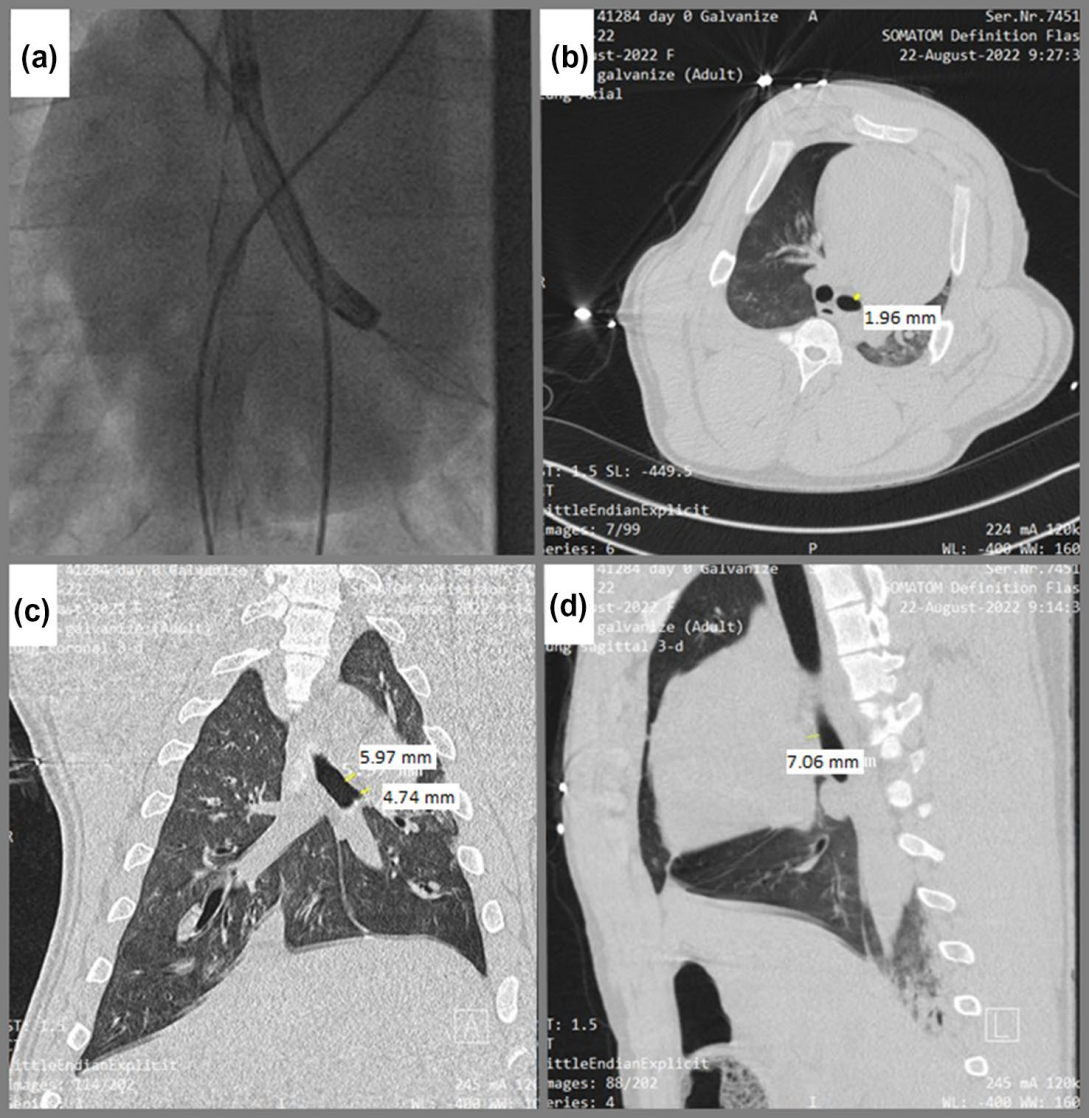
(**a**) Fluoroscopic image of bronchoscope in the left main bronchus of the swine with deployment of the basket electrode (**b-d**) Representative swine CT scans are provided with proximity to the epicardial surface measured in the axial (**b**, 1.96 mm), coronal (**c**, 5.97 mm and 4.74 mm), and sagittal (**d**, 7.06 mm) planes

The minimum distance between the heart and mainstem bronchi where the basket electrode was placed was measured by combining a fluoroscopic image of basket deployment with CT images of the porcine chest indicated a distance as close as 2 mm (Fig. 4).

**Fig. 5:**
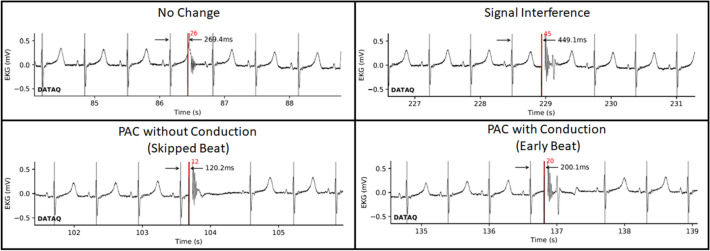
Examples of cardiac effects from asynchronous PEF delivery. There may be no change (top left), an artifactual change in the immediate heartbeat ECG due to signal interference (top right), a PAC without ventricular conduction that briefly delays the subsequent heartbeat (bottom left), or a PAC that conducts through to the ventricle resulting in an early heartbeat (bottom right). The two PAC conditions are seen to alter that specific R–R interval but have no residual effect beyond the subsequent heartbeat. Black arrows and numbers denote the time interval from QRS complex to PEF delivery. Red bars and numbers denote when the PEF packet was delivered and the PEF packet number

A total of 3,125 PEF packets were delivered to four pigs across the three studies. No sustained changes to the cardiac rhythm nor to the ECG waveform were noted, particularly no atrial nor ventricular fibrillation, no ST elevations, and no ventricular tachycardia. Overall, the cardiac response to PEF delivery fell into one of four categories, which are shown in Fig. 5:

1. *None:* No effect or change to the ECG resulting from the packet delivery.
2. *Signal Interference:* PEF delivery resulted in ECG artifact. When this artifact coincided with an ECG feature (e.g., QRS complex) the feature appearance was altered. There is no impact to cardiac activation or timing and is an artifact of the experimental setup and data-recording devices.
3. *Premature-atrial contraction (PAC) without ventricular conduction:* The delivered packet stimulates an atrial contraction prior to the native conducted beat from the sinoatrial (SA) node. The atrioventricular (AV) node is refractory during this premature beat, and thus, the electrical signal does not conduct to capture the ventricles. The SA node, however, has been reset, and thus, a subsequent normal heartbeat does not occur until a period following the premature-atrial contraction that aligns with the basal heart rate. This manifests on an ECG as a ‘non-conducted,’ or skipped, beat with a cycle length approximately double the basal cycle length. The cardiac rhythm returns to the basal heart rate following the subsequent heartbeat.
4. *Premature-atrial contraction (PAC) with ventricular conduction:* The delivered packet stimulates an atrial contraction prior to the native conducted beat from the SA node. The AV node is not refractory and permits the premature beat to conduct and activate the ventricles, resulting in normal ventricular conduction. This manifests as a premature heartbeat that occurs prior to when the basal heartbeat would be encountered. Following the premature heartbeat, the cardiac rhythm returns to its basal rate, which at times, is encountered with either sinus reset or with continued sinus timing.

A summary of treatments delivered, and the adjudicated review of the cardiac rhythms and ECG waveforms, are provided in Table 1. There were no instances of sustained changes to the ECG waveform (e.g., no ST elevation) or to cardiac rhythm for any PEF deliveries, including no episodes of bradycardia, tachycardia, atrial fibrillation, or ventricle fibrillation. Furthermore, findings were limited to single PACs. These data are further delineated by lung and location in Supplemental Table 1. Brief descriptions of the individual experiment results are provided below.

**Table 1:** Summary of PEF delivery

Focus	R-Trigger delay range (ms)	R-trigger delay resolution (ms)	Total ECG sweeps	Total packets	Total PAC observations	Bradycardia	Tachycardia	Atrial fibrillation	Ventricle Fibrillation	ST elevation
ECG Sweep	10-R*	10	16	1174	186	0	0	0	0	0
Specific T-Wave Triggers	100–490	10	4	160	46	0	0	0	0	0
Multiple Packets	40-R*	40	22	1610	71	0	0	0	0	0

*When complete ECG sweeps were performed, the R-wave trigger delay ranged from the shortest resolution timepoint until the subsequent R-wave. This varied based on pig heart rate

#### Full ECG Sweep

A total of 1,180 packets were delivered across the two pigs. Each section (distal and proximal) of both the left and right lungs received a full cardiac cycle PEF sweep, resulting in 16 total complete cardiac cycle PEF sweeps. Only PACs occurred, as described previously, and only with the basket electrode in the proximal mainstem bronchi. When a PAC occurred, its impact was only on the timing of the immediate subsequent heartbeat, and this spontaneously resolved immediately in all cases, regardless of whether it was conducted to the ventricles or not. Overall, there were no safety risks, as defined by any arrhythmias, any aberrant ventricular conduction outside of the normal conducting system, or any changes to the ST segment, etc., associated with the packet delivery.

#### High-Resolution T-Wave Delivery

During the high-resolution T-wave delivery, a single pig received 160 packets at the clinical PEF dose. Overall, despite the high-resolution delivery of PEFs across the most vulnerable portion of the ECG, no durable (> 1 heartbeat) changes to cardiac rhythm were observed, and no appreciable safety risk associated with the biphasic PEF packet delivery in any region was noted.

#### Multiple Packets

In all, 1,945 packets were delivered over 341 PEF activations to a single pig. PACs were noted, again only with the basket electrode in the proximal left mainstem bronchus when the treatment was delivered sufficiently beyond the refractory period. A summary of observations is provided in Table 2. Consistent with the prior two experiments, there were no observed incidences of durable changes to cardiac rhythm, including incidences of fibrillation or other dangerous arrhythmias. These findings were consistent regardless of the PEF packet delivery rate, whether 5 or 10 packets were delivered, and without regard for the initial timing of the first packet delivered. A discrete breakdown of the experimental conditions and PAC induction are in Supplemental Table 2.

**Table 2:** Summary of experimental results for multi-packet PEF delivery to the heart

Set	Total Sets	Packets per Set	Delivery Rate	No effect	PAC without conduction	PAC with conduction
1	90	5	5 Hz	5	17	5
2	95	5	ECG	59	5	10
3	89	5	0.66 Hz	32	1	21
4	29	10	5 Hz	0	6	5
5	9	10	ECG	6	0	0
6	10	10	0.66 Hz	0	0	1

All animals in these studies had normal cardiac rhythms immediately post-treatment that lasted until their pre-specified survival timepoints. Therefore, these data demonstrate the absence of any clinically meaningful cardiac arrythmias regardless of test conditions for electrode location, PEF delivery relative to the cardiac rhythm, nor number of subsequent packets.

## Discussion

A systematic exploration of the arrhythmogenic potential of a biphasic, monopolar PEF system in the airways was performed. A numerical simulation evaluated the likelihood of arrythmia induction as a function of pulse duration, applied voltage, and distance to the heart. Experimentally, individual packet dosing and waveform characteristics were matched to that of treatment parameters for clinical airway therapy for both single- and multiple-packet treatments.

No sustained changes to the ECG waveform or to the cardiac rhythm were observed despite the increasing vigor of the various attempts to induce an arrhythmia for the short-duration (≤ 10 µs) biphasic pulses tested in this experimental setup. These data are in stark contrast to results previously reported for a different PEF technology by Deodhar et al. [5]. In that study, 90 NanoKnife (AngioDynamics, NY, USA) pulses, each 70 µs pulse duration, were delivered over a range of voltages and locations relative to the heart. They observed a large proportion of the test conditions induced a range of cardiac arrhythmias, including ventricular tachycardia and ventricular fibrillation when energy was delivered asynchronously. When R-trigger cardiac synchronization was included according to their recommendations, ST elevation and T-wave inversions were still observed. These differences underscore that not all PEF technologies are alike, with key distinctions in terms of safety and efficacy for various electric field platforms and delivery systems. There are other differences between this study and the Deodhar study beyond pulse duration, including the electrode arrangement (monopolar here), and the biphasic waveform.

In this study, the end-effector was purposely placed in the closest airway positions to the heart (approximately 2 mm from cardiac muscle [13]. Pigs are documented to be significantly more arrhythmogenic than humans [34]. Despite these worst-case conditions, the only alteration to the ECG was occurrence of PACs when energy was delivered with the basket electrode in the proximal mainstem bronchi. Since the arrhythmogenicity of PEF energy decreases dramatically for biphasic waveforms, such as the one used for the PEF therapy evaluated here. These data are in agreement with the suggestion by Siddiqui et al. that biphasic PEF may obviate the need for cardiac synchronization [27].

Theoretical examination of cardiac activation and fibrillation thresholds demonstrated that activation is likely for short (< 10 µs) individual monophasic pulses even at low PEF applied voltages (≤ 1 kV). However, fibrillation thresholds are approximately two orders of magnitude greater, and thus, induction of arrythmia was only noted when the pulse duration was 50 µs or longer, with 100 µs pulses able to induce fibrillation for all examined voltages. Notably, in most published biphasic PEF waveforms, the pulse duration for each phase is 0.5 to 5 µs, falling well within this ‘zone of safety’ for monopolar electrodes and applied voltages well above 3.0 kV. This is consistent with the conditions of the commercially available PEF system experimentally evaluated here for inducing arrythmia in pigs. The system used a PEF protocol that encompassed voltage and frequency characteristics consistent with those evaluated in the simulation, and were shown to cause cardiac activation, but not fibrillation under any of the conditions tested.

One limitation of the theoretical portion of this study is that it evaluated arrythmia risk for a single monophasic pulse. While this is consistent with some commercial PEF systems that use a series of long monophasic pulses (50–100 µs duration), it does not fully recapitulate the cardiac effects potentially induced by a biphasic waveform comprising a compilation of short pulses (0.5–5 µs) of alternating phase in rapid succession. This limitation is a result of the previous relevant explorations on activation and fibrillation risk for cardiac tissue as a function of pulse waveform characteristics. Future work should better characterize the likelihood of activation and fibrillation induction under typical PEF ablation waveforms that incorporate these additional variables (biphasic pulse width, number of biphasic cycles comprising a complete packet, and delivery rate of multiple biphasic packets).

Notably, the decay of electric voltage for monopolar versus bipolar electrode arrangements follows different patterns, and thus, different electrode geometries should undergo additional investigation when pulse duration exceeds 10 µs. However, despite the different electrode geometry systems, the findings from this theoretical investigation closely match the observations of Deodhar et al. [5]. In that study, fibrillation was encountered when the electrodes were within 1.7 cm of the heart for 70 µs pulses, even at only 1.6 kV delivered [5]. The theoretical section of this study predicts a fibrillation threshold of 1.1 kV for 70 µs pulses. The numerical simulation showed a 1.6 kV pulse would exceed that threshold at approximately 1.1 cm. The consistent theoretical results with the *in vivo* empirical data bolsters the theoretical simulation. This supports the inability for waveforms with ≤ 10 µs pulse duration to induce cardiac arrythmias until voltages exceed 5.0 kV.

The PEF therapy protocol evaluated experimentally is capable of therapeutic cell death. The protocol tested was optimized to produce significant epithelial (43 ± 27%) and submucosal gland (3 ± 4%) cell death without causing changes to airway integrity or function (data unpublished). The clinical utility of the protocol used by this system was evaluated in a multicenter clinical trial [6, 31], where patients with chronic bronchitis (CB) were treated. No device-related adverse events were found, while patient-reported symptom improvements of − 8.0 (*p* < 0.001) and − 14.7 (*p* < 0.001) were observed for the chronic obstructive pulmonary disease assessment test (CAT) and St. George’s Respiratory Questionnaire (SGRQ), respectively. These changes are well beyond the minimally clinically important differences in each test (− 2 and − 4 points, respectively). Tissue measures of goblet cell hyperplasia were reduced by roughly 39% (*p* < 0.001) when comparing pretreatment with post-treatment airway tissue samples.

The occasional induction of PACs was the only noted cardiac impact from the delivery of PEF in these studies and is consistent with the simulated prediction of cardiac stimulation but not arrhythmia induction. PACs are a common clinical phenomenon, occurring at least once per 24 h period in > 99% of the general adult population [4]. The impact of the delivered energy was limited to only a single beat, never extending to any subsequent beats. Therefore, these findings do not demonstrate a measurable risk to patient health.

One study consideration is that the relatively healthy porcine hearts could be more resilient compared to human hearts with underlying disease states, such as myocardial ischemia. However, it should be noted that Walcott et al. showed that swine are nearly three times more sensitive to electrically induced arrhythmia than humans [34], and thus, serve as a highly sensitive model for cardiac safety. This study also relied on historic literature to demonstrate the arrhythmogenic potential of longer duration monophasic waveforms rather than a positive control [5]. Finally, while it is possible that humans and pigs have different responses to monophasic versus biphasic waveforms, this is unlikely since both exhibit the same preferential response for biphasic versus monophasic cardioversion [26].

This experimental investigation used a specific waveform and device configuration for a commercially available clinical system. The stochastic nature of arrythmias required comprehensive testing to determine the likelihood of arrythmia for this technology. Other technologies would require a similarly comprehensive evaluation overall. Variables including different electrode geometries, biphasic waveforms, voltages, and proximities to the heart, may be used collectively to establish a risk profile for when cardiac synchronization is warranted versus unnecessary.

This study theoretically evaluated several relevant variables to cause arrhythmia, including applied voltage, pulse duration, and proximity to the heart. It experimentally evaluated a biphasic, monopolar, PEF technology for treating the airways to determine the safety of asynchronous PEF delivery throughout various regions of the ECG waveform. Despite the delivery of thousands of packets, including many directly over the vulnerable T-wave, only occasional PACs were observed, with no cardiac arrhythmias or changes to ECG waveform. This study demonstrates that appropriately established PEF parameters, such as those used in the clinical RheOx system, do not appear to require cardiac synchronization of energy delivery to prevent concerning arrhythmias.

### Supplementary Information

Below is the link to the electronic supplementary material.

(Top) Simulation tissue electrical conductivity as a function of electric field exposure. (Bottom) Simulated electric current and impedance as a function of simulation applied voltages for 500 to 5000 V. Supplementary file1 (TIF 1480 kb)

(A) Data from Lawo et al. (B) Power law curve fitting for the “Stimulation (coil)” curve from (A). (C) Power law curve fitting for the “Induction” curve from (A). Note: “Stimulation (coil)” was used for stimulation since it reflects the same electrode relationship that was used to generate the “Induction” data. Supplementary file2 (TIF 1931 kb)

Supplementary file3 (DOCX 18 kb)

Supplementary file4 (DOCX 14 kb)
