# Supplementary figures and images for: Absence of Arrhythmogenicity with Biphasic Pulsed Electric Fields Delivered to Porcine Airways
Source: Ann Biomed Eng. 2023 Apr 25;52(1):1–11. doi: 10.1007/s10439-023-03190-5 (PMC10761461; doi:10.1007/s10439-023-03190-5)

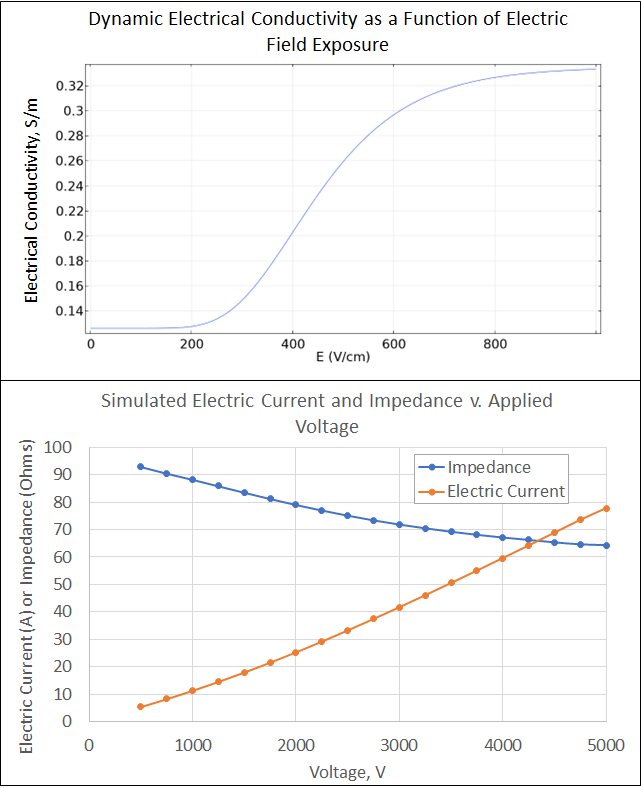

Supplement: Supplementary file 1 — (Top) Simulation tissue electrical conductivity as a function of electric field exposure. (Bottom) Simulated electric current and impedance as a function of simulation applied voltages for 500 to 5000 V. Supplementary file1 (TIF 1480 kb) [file 10439_2023_3190_MOESM1_ESM.tif]

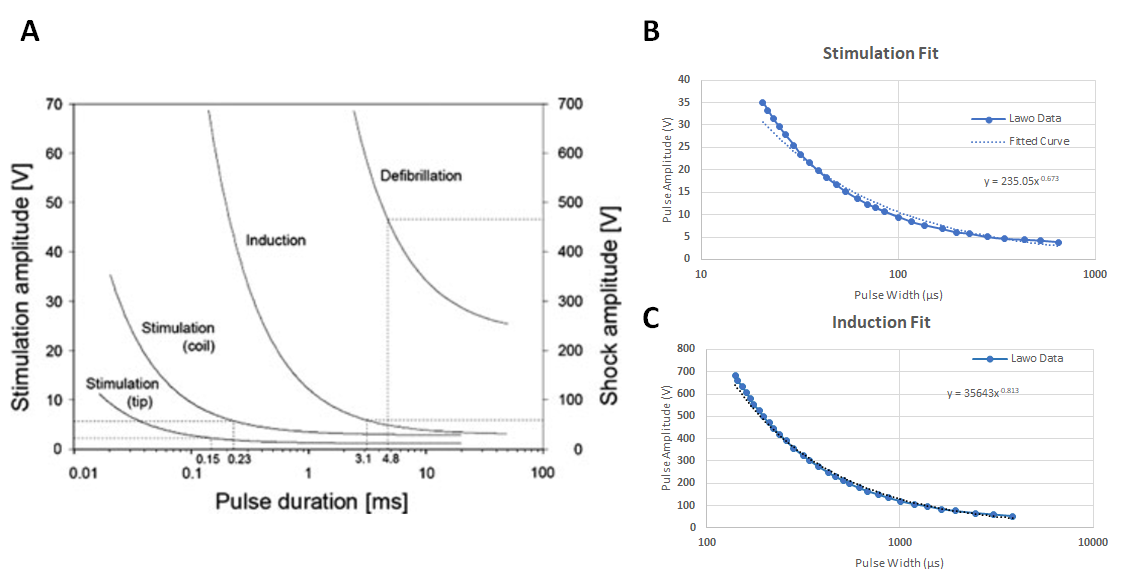

Supplement: Supplementary file 2 — (A) Data from Lawo et al. (B) Power law curve fitting for the “Stimulation (coil)” curve from (A). (C) Power law curve fitting for the “Induction” curve from (A). Note: “Stimulation (coil)” was used for stimulation since it reflects the same electrode relationship that was used to generate the “Induction” data. Supplementary file2 (TIF 1931 kb) [file 10439_2023_3190_MOESM2_ESM.tif]
