# Supplementary material for: Absence of Arrhythmogenicity with Biphasic Pulsed Electric Fields Delivered to Porcine Airways
Source: Ann Biomed Eng. 2023 Apr 25;52(1):1–11. doi: 10.1007/s10439-023-03190-5 (PMC10761461; doi:10.1007/s10439-023-03190-5)
Supplement: Supplementary file 3 — Supplementary file3 (DOCX 18 kb) [file 10439_2023_3190_MOESM3_ESM.docx]

Supplemental Table 1

Table 1: Summary of PEF Delivery

| **Pigs** | **Focus** | **Lung** | **Section** | **R-Trigger Delay Range, ms** | **R-Trigger Delay Resolution, ms** | **Packet Count per Activation** | **Total ECG Sweeps** | **Total Packets** | **Total PAC Observations*** | **Brady-cardia** | **Tachy-cardia** | **Atrial Fibrillation** | **Ventricle Fibrillation** | **ST Elevation** |
| --- | --- | --- | --- | --- | --- | --- | --- | --- | --- | --- | --- | --- | --- | --- |
| **2** | ECG Sweep | Left | Distal | 10-R* | 10 | 1 | 2 | 127 | 0 | 0 | 0 | 0 | 0 | 0 |
|  |  | Left | Proximal | 10-R* | 10 | 1 | 2 | 117 | 23/7 | 0 | 0 | 0 | 0 | 0 |
|  |  | Right | Distal | 10-R* | 10 | 1 | 2 | 128 | 0 | 0 | 0 | 0 | 0 | 0 |
|  |  | Right | Proximal | 10-R* | 10 | 1 | 2 | 122 | 0 | 0 | 0 | 0 | 0 | 0 |
| **2** | ECG Sweep | Left | Distal | 10-R* | 10 | 1 | 2 | 166 | 0 | 0 | 0 | 0 | 0 | 0 |
|  |  | Left | Proximal | 10-R* | 10 | 1 | 2 | 168 | 71/31 | 0 | 0 | 0 | 0 | 0 |
|  |  | Right | Distal | 10-R* | 10 | 1 | 2 | 177 | 0 | 0 | 0 | 0 | 0 | 0 |
|  |  | Right | Proximal | 10-R* | 10 | 1 | 2 | 169 | 44/10 | 0 | 0 | 0 | 0 | 0 |
| **3** | Specific T-Wave Triggers | Left | Distal | 100-490 | 10 | 1 | 1 | 40 | 0 | 0 | 0 | 0 | 0 | 0 |
|  |  | Left | Proximal | 100-490 | 10 | 1 | 1 | 40 | 0/46 | 0 | 0 | 0 | 0 | 0 |
|  |  | Right | Distal | 100-490 | 10 | 1 | 1 | 40 | 0 | 0 | 0 | 0 | 0 | 0 |
|  |  | Right | Proximal | 100-490 | 10 | 1 | 1 | 40 | 0 | 0 | 0 | 0 | 0 | 0 |
| **4** | Multiple Packets | Left | Distal | 40-R* | 40 | 5 @ 300 ppm | 22 | 110 | 0 | 0 | 0 | 0 | 0 | 0 |
|  |  |  |  |  |  | 5 @ 40 ppm | 22 | 110 | 0 | 0 | 0 | 0 | 0 | 0 |
|  |  |  |  |  |  | 5 @ 70 ppm | 22 | 110 | 0 | 0 | 0 | 0 | 0 | 0 |
|  |  | Left | Proximal | 40-R* | 40 | 5 @ 300 ppm | 23 | 115 | 17/5 | 0 | 0 | 0 | 0 | 0 |
|  |  |  |  |  |  | 5 @ 40 ppm | 22 | 110 | 5/10 | 0 | 0 | 0 | 0 | 0 |
|  |  |  |  |  |  | 5 @ 70 ppm | 21 | 105 | 1/21 | 0 | 0 | 0 | 0 | 0 |
|  |  |  |  |  | 40 | 10 @ 300 ppm | 29 | 145 | 6/5 | 0 | 0 | 0 | 0 | 0 |
|  |  |  |  |  |  | 10 @ 40 ppm | 9 | 45 | 0 | 0 | 0 | 0 | 0 | 0 |
|  |  |  |  |  |  | 10 @ 70 ppm | 10 | 50 | 0/1 | 0 | 0 | 0 | 0 | 0 |
|  |  | Right | Distal | 40-R* | 40 | 5 @ 300 ppm | 23 | 115 | 0 | 0 | 0 | 0 | 0 | 0 |
|  |  |  |  |  |  | 5 @ 40 ppm | 29 | 145 | 0 | 0 | 0 | 0 | 0 | 0 |
|  |  |  |  |  |  | 5 @ 70 ppm | 24 | 120 | 0 | 0 | 0 | 0 | 0 | 0 |
|  |  | Right | Proximal | 40-R* | 40 | 5 @ 300 ppm | 22 | 110 | 0 | 0 | 0 | 0 | 0 | 0 |
|  |  |  |  |  |  | 5 @ 40 ppm | 22 | 110 | 0 | 0 | 0 | 0 | 0 | 0 |
|  |  |  |  |  |  | 5 @ 70 ppm | 22 | 110 | 0 | 0 | 0 | 0 | 0 | 0 |

*Results presented as ”With conduction / without conduction”
