# Supplementary material for: Absence of Arrhythmogenicity with Biphasic Pulsed Electric Fields Delivered to Porcine Airways
Source: Ann Biomed Eng. 2023 Apr 25;52(1):1–11. doi: 10.1007/s10439-023-03190-5 (PMC10761461; doi:10.1007/s10439-023-03190-5)
Supplement: Supplementary file 4 — Supplementary file4 (DOCX 14 kb) [file 10439_2023_3190_MOESM4_ESM.docx]

Supplemental Table 2: Summary of experimental results for multi-packet PEF delivery to the heart

| **Set** | **Total Sets** | **Lung** | **Location** | **Packets** | **Delivery Rate** | **No effect** | **PAC without conduction** | **PAC with conduction** |
| --- | --- | --- | --- | --- | --- | --- | --- | --- |
| **1** | 23 | Right | Distal | 5 | 5 Hz | 3 | 0 | 0 |
| **2** | 29 | Right | Distal | 5 | ECG | 29 | 0 | 0 |
| **3** | 24 | Right | Distal | 5 | 0.66 Hz | 24 | 0 | 0 |
| **4** | 22 | Right | Proximal | 5 | 5 Hz | 0 | 0 | 0 |
| **5** | 22 | Right | Proximal | 5 | ECG, 900 ms | 14 | 0 | 0 |
| **6** | 22 | Right | Proximal | 5 | 0.66 Hz | 4 | 0 | 0 |
| **7** | 19 | Right | Proximal | 5 | ECG, 850 ms | 7 | 0 | 0 |
| **8** | 22 | Left | Distal | 5 | 5 Hz | 2 | 0 | 0 |
| **9** | 22 | Left | Distal | 5 | ECG, 900 ms | 15 | 0 | 0 |
| **10** | 22 | Left | Distal | 5 | 0.66 Hz | 4 | 0 | 0 |
| **11** | 23 | Left | Proximal | 5 | 5 Hz | 0 | 17 | 5 |
| **12** | 22 | Left | Proximal | 5 | ECG, 900 ms | 1 | 5 | 10 |
| **13** | 21 | Left | Proximal | 5 | 0.66 Hz | 0 | 1 | 21 |
| **14** | 29 | Left | Proximal | 10 | 5 Hz | 0 | 6 | 5 |
| **15*** | 9 | Left | Proximal | 10 | ECG, 850 ms | 6 | 0 | 0 |
| **16*** | 10 | Left | Proximal | 10 | 0.66 Hz | 0 | 0 | 1 |

*Packet bundle start resolution of 80 ms, instead of the 40 ms used in the other trials.
